# Cutaneous Leishmaniasis in the Context of Global Travel, Migration, Refugee Populations, and Humanitarian Crises

**DOI:** 10.3390/clinpract15040077

**Published:** 2025-04-08

**Authors:** Janice Kim, Tarek Zieneldien, Sophia Ma, Bernard A. Cohen

**Affiliations:** 1 College of Osteopathic Medicine, Michigan State University, East Lansing, MI 48824, USA; 2 School of Medicine, Johns Hopkins University, Baltimore, MD 21205, USA; 3 Department of Dermatology, The Johns Hopkins Hospital, Baltimore, MD 21287, USA

**Keywords:** cutaneous leishmaniasis, global health, refugee health

## Abstract

Cutaneous leishmaniasis (CL) is a vector-borne infection caused by protozoan parasites belonging to the genus *Leishmania*. CL is an emerging global health concern due to increasing migration, travel, and climate change. Traditionally, it was confined to endemic regions such as the Americas, the Middle East, and Central Asia; however, it is now spreading to non-endemic areas. Climate change has further contributed to the expansion of sandfly habitats, increasing CL transmission risk in previously unaffected areas. Healthcare providers in non-endemic regions often misdiagnose CL, delaying treatment and morbidity. Diagnosis remains challenging due to the need for species-specific identification, while treatment is limited by cost, availability, and personnel expertise. This review explores the epidemiology, clinical presentation, diagnostic challenges, and management of CL in the context of global mobility. It highlights rising CL cases in refugee settlements, particularly in Lebanon and Jordan, due to poor living conditions, inadequate vector control, and healthcare barriers. While there have been advances in systemic and topical therapies, access in refugee and resource-poor settings remains a barrier. Addressing the global burden of CL requires improved surveillance, healthcare provider training, and increased awareness. By enhancing global collaboration and policy changes, public health efforts can mitigate the expanding impact of CL.

## 1. Introduction

Leishmaniasis is caused by the protozoan parasite from the genus *Leishmania*, and is transmitted through the bite of infected female sandflies [1]. Leishmaniasis presents in three main forms: visceral, which is fatal if untreated and characterized by irregular fever, weight loss, splenomegaly, hepatomegaly, and anemia; cutaneous, the most common, causing skin ulcers that leave scars and lead to disability; and mucocutaneous, which can destroy the mucous membranes of the nose, mouth, and throat [2]. Cutaneous leishmaniasis (CL) has become increasingly relevant due to global travel, migration, and climate change, as these factors have contributed to its spread beyond traditionally endemic regions such as in tropical and subtropical regions [3]. The intersection of dermatological conditions, global migration, and travel medicine has become more significant, as studies have shown that skin diseases are among the most common health concerns affecting adult refugees in the United States [4]. The rising incidence of CL is evident from recent outbreaks among Syrian refugees, particularly in settlements such as Lebanon and Jordan [5]. These outbreaks have been driven by delayed diagnosis and treatment, due to limited healthcare access, language barriers, and a lack of awareness among healthcare providers in non-endemic countries, highlighting the urgent need for global healthcare systems to improve awareness and response to dermatologic diseases [6]. Additionally, the rise in imported cases of CL from Mexico to the United States underscores the role of travel-related disease transmission [7,8]. This review explores the epidemiology, clinical presentation, diagnostic challenges, and management of CL in the context of global mobility, emphasizing the lack of familiarity with this disease among health practitioners in non-endemic areas and the growing challenges associated with it due to its increasing incidence in North America and Europe.

## 2. The Impact of Refugee Settlements on the Spread, Diagnosis, and Treatment of Cutaneous Leishmaniasis

With increasing globalization, travel, and forced migration, CL, which was once confined to specific endemic regions, is now emerging as a global health concern [3]. CL is endemic in the Americas, the Mediterranean basin, the Middle East, and Central Asia, but displaced refugees have inadvertently introduced CL to regions previously unaffected by the disease, leading to new outbreaks and increased public health challenges [5]. Forced displacement due to armed conflicts and political instability has led to mass migrations, and as a result, refugee settlements in Lebanon and Jordan have experienced increased transmission of CL, as it is endemic in Syria [9]. The disease, transmitted by infected female sandflies, spreads rapidly in overcrowded camps, where poor sanitation, limited healthcare, and environmental conditions favor sandfly breeding [10]. Syrian refugees reside in poorly made camps and tents, making them vulnerable to environmental conditions and lacking adequate protection against sandfly bites, especially during the dry summer months when the sandfly population increases, heightening the risk of exposure and transmission [11]. Additionally, due to the high population density in refugee settlements, there is poor waste management and open sewage that attracts rodent reservoirs, further sustaining the transmission cycle [5].

Pre-conflict, CL prevalence in Syria was restricted to two areas, Aleppo and Damascus, with an incidence of 23,000 cases/year [12]. However, following the conflict in early 2013, the cases increased to 41,000 and spread to different regions in Syria due to the breakdown of healthcare systems and vector control programs, exacerbating the CL situation among refugees [12]. From 2005 to 2011, the annual number of CL cases recorded in Lebanon ranged from 0 to 6 cases [10]. In 2012, after the Syrian crisis, the number increased to 1275 cases. The numbers slowly increased, with a peak of 1383 cases in 2014 [10]. Additionally, in Turkey, non-endemic parasite strains such as *L. major* and *L. donovani* were introduced by incoming Syrian refugees [13]. Refugees frequently experience delayed diagnosis and treatment due to financial constraint, language barriers, and lack of resources, allowing infections to progress [14]. In many of these non-endemic areas, healthcare providers also may lack awareness and experience in diagnosing CL, leading to misdiagnosis and inappropriate treatment [15]. A German study found that 40% of patients from Syria reported treatment delays of three months or more after arriving in Germany, with some waiting 32 months, due to the unfamiliarity of healthcare providers with CL and its tendency to mimic other skin conditions such as lupus vulgaris, lupus erythematosus, sarcoidosis, and granuloma annulare, leading to misdiagnosis [16]. Moreover, the study found that 85% of Syrian refugees with CL had complex lesions, often due to previous treatment failures or facial localization [16]. CL can lead to significant scarring and, if occurring on the face, can lead to social stigma and psychological distress among refugees already suffering from the trauma of displacement from their home country [17].

The treatment of CL in refugee populations presents significant challenges due to the complexity of disease management, often requiring systemic treatments and repeated systemic treatments [5]. These systemic treatments require consistent medical supervision, access to specialized drugs, and prolonged follow-up, which are resources that are limited in refugee settlements [18]. Moreover, the instability of these settings, combined with the limited healthcare infrastructure and financial constraints, make effective treatment extremely challenging [18]. Without global efforts to improve access to healthcare and vector control, CL will continue to cause significant morbidity and disability in vulnerable displaced populations.

## 3. Migrant Populations, Dermatological Issues, and Healthcare

Migrants, refugees, and asylum seekers face unique dermatologic challenges due to poor living conditions, limited healthcare access, and cultural barriers. As of mid-2024, it had been reported that nearly 122.6 million individuals were displaced globally, with more than 69% of refugees residing in neighboring countries, and approximately 71% of refugees living in low- and middle-income countries (LMICs) [19]. As reported by the United Nations High Commissioner for Refugees, approximately 6.6 million refugees inhabit camps, with the vast majority living in informal settlements or urban areas [20].

Often, migrants experience deprivation during their journey, facing poor living conditions and inadequate hygiene, residing in damp and unsanitary conditions that promote the spread of viruses and bacteria. These individuals are forced to overlook their health as they prioritize basic needs such as finding shelter, food, and water [21]. Due to these conditions, many individuals are at risk of skin diseases such as scabies, acute dermatitis, skin ulcers, and lice. A study on the dermatologic conditions of adult refugees resettling in the United States found that 53.5% of participants were diagnosed with a dermatologic condition within one month [4]. Consequently, anxiety, post-traumatic stress disorder, and depression are present in high rates among these vulnerable populations [22,23]. They also experience a variety of post-migration stressors, such as social isolation, family separation, housing insecurity, and financial instability [24]. These stressors in turn worsen conditions such as atopic dermatitis, as emotional stress and anxiety have been found to exacerbate itching and trigger the release of inflammatory mediators in patients with atopic dermatitis [25,26].

In humanitarian crises, where resources are primarily allocated toward basic medical care, diagnostic testing for dermatologic disorders is limited. Thus, most dermatologic diagnoses are made based on clinical evaluation [27,28]. Besides this, additional barriers exist to healthcare access, including lack of access to dermatologists and mental health services, movement across and within international borders, legal and administrative barriers, armed conflicts that affect access to resources and security, and language differences [18]. Moreover, navigating foreign healthcare systems with limited financial resources also contributes to the decreased quality of care that displaced individuals receive [29]. To address these disparities, the United Nations High Commissioner for Refugees focuses on reducing epidemic morbidity, enhancing childhood survival, delivering primary care, preventing and containing noncommunicable diseases, integration into national health services, developing action plans with a focus on nutrition screening and immunization, and rationalizing specialty care access during emergency situations [18,30]. Despite these initiatives, the provision of dermatologic care to displaced people and migrants remains insufficient, resulting in the need for greater advocacy to ensure equitable access to skin health services.

## 4. Migration, Climate Change, and Travel in Emerging Disease Patterns of CL

The prevalence of dermatologic conditions among migrants and travelers is a growing health concern, with studies indicating that skin diseases are among the most common conditions affecting displaced populations [4]. A study found that 39% of adult refugees in the United States had at least one dermatologic condition within a year of resettlement, highlighting the significant burden of skin diseases in this population [4]. Migrants often face multiple risk factors such as poor sanitation, inadequate access to healthcare, malnutrition, and exposure to new environmental pathogens, which all contribute to a high frequency of infectious and inflammatory skin conditions [31]. Many healthcare providers in the U.S. and Europe lack experience diagnosing and managing CL, leading to misdiagnosis or treatment delays [32]. There is a growing need for increased awareness among healthcare providers about dermatologic conditions, because early diagnosis and treatment are crucial amid rising global mobility and the emergence of new CL cases in non-endemic areas.

In 2023, there was a notable increase in imported cases of CL from Mexico that was observed in Europe. A case series reported that six travelers who returned from Mexico had CL, which was a significant rise compared to the previous two decades [7]. Additionally, from March to August 2023, it was also found that three additional unrelated cases were diagnosed in Berlin, Germany, all linked to exposure in southeastern Mexico, where the *L. mexicana* strain is endemic to the Yucatan peninsula [7]. The recent surge in imported cases of CL can be attributed to increased travel to endemic regions and environmental changes such as climate change, which have altered the habitats of sandfly vectors responsible for transmitting *Leishmania* parasites [33]. Rainfall, temperature fluctuations, and deforestation have influenced the distribution and population density of these vectors, which have expanded their range and increased transmission in previously unaffected areas, affecting both migrant populations and international travelers [34]. With rising temperatures, sandflies have been able to survive at higher altitudes, leading to the spread of CL into new areas, including Europe and North America, while deforestation and agricultural expansion have increased human–sandfly interactions, further increasing transmission risk [35]. In one study, it was found that there were significant correlations between leishmaniasis incidence and climate parameters, with a positive correlation seen between incidence and precipitation [36]. Additionally, a study conducted in Sri Lanka examining the impact of climate and land use on sand fly density found that climate is the primary determinant, with rainfall, temperature, and sunshine hours playing the most significant roles in increasing sandfly populations across the country [37]. Moreover, a study conducted in Morocco found that rising temperatures and changing rainfall patterns will create more suitable habitats for the *Leishmania tropica* sand fly vector, increasing the risk of CL in the future and leading to a wider geographical spread and prolonged transmission periods [38]. As migration and travel facilitate disease transmission, improving awareness will be essential in mitigating the global burden of CL. The rising prevalence of dermatologic conditions among migrants and travelers, along with the increasing incidence of imported CL, highlights the growing impact of climate change and infectious diseases patterns that drive the spread of CL into new regions.

## 5. Mechanisms and Differences in Presentation

CL is caused by various species of Leishmania parasites, which are transmitted through the bite of infected female sandflies from the genera *Phlebotomus* and *Lutzomyia* [39]. After a sandfly bite, *Leishmania* promastigotes enter the dermis and are rapidly phagocytosed by macrophages. They then evade the complement system by using host receptors, while inhibiting membrane attack complex formation. This allows them to survive and replicate within host cells, transforming into intracellular amastigotes [40]. In an immune response, the balance between Th1 and Th2 response is crucial for infection control [41]. However, in CL, an imbalance in the immune response drives disease pathology, with a Th1-dominant response controlling the parasite but causing skin lesions, while a Th2-dominant response leads to parasite persistence, chronic lesions, or diffuse disease forms [41].

The most common manifestation of leishmaniasis is localized cutaneous leishmaniasis (LCL), and is typically caused by *L. major*, *L. tropica*, and *L. aethiopica* [1]. LCL is characterized by the development of slow-healing skin lesions that develop from small red papules into painless nodules, before forming circumscribed ulcers at or near the site of an infected sandfly bite [42]. The staphylococcal species are the most common cause of secondary infections, often leading to misdiagnosis as other infectious skin lesions [43]. The onset of clinical symptoms ranges from two to eight weeks, and the infection sites are usually found on the arms, legs, and face. The lesions self-heal without treatment; however, the use of antimonials and amphotericin B have cure rates of greater than 90% [44].

Diffuse CL is caused by *L. aethiopica*, *L amazonesis*, and *L. mexicana* [45]. It is prevalent in immunocompromised patients and the infection begins as a papule on the extremities or face, and spreads slowly, leading to the further development of papules [46]. This form is highly resistant to treatment and persists for many years, leading to uncontrollable parasite dissemination [47]. Patients who are infected have the Th2-dominant response and fail to mount a delayed-type hypersensitivity response, resulting in uncontrolled parasite replication inside macrophages [48].

## 6. Challenges in Diagnosis and Management

Managing leishmaniasis remains a challenge for physicians in previously non-endemic areas, often resulting in delayed diagnosis, misdiagnoses, and inadequate treatment regimens, especially in cases that only manifest cutaneously [32]. In general, differentiating between CL and skin cancers can be difficult due to a variety of factors. Leishmania parasites proliferate within antigen-presenting myeloid cells such as monocytes, macrophages, and dendritic cells, leading to the formation of macrophage clusters and generating a strong inflammatory reaction because of their high antigenic burden [49]. IFN-γ is primarily produced by CD4 T cells, while CD8 T cells and natural killer cells also contribute to its production. IFN-γ activates macrophages for parasitic control, but excessive parasitic loads or weak responses can lead to tissue destruction and chronic lesions [50]. Even though T cells and macrophages inhibit tumorigenesis early, continuous inflammation could drive tumor progression. Thus, chronic immune activation by Leishmania could produce a microenvironment that encourages tumor formation [32,51]. Studies have shown that in non-endemic areas, such as Northern Europe, CL is sometimes misdiagnosed as skin cancer, and other conditions such as follicular cysts, sarcoidosis, lymphoma, epithelial neoplasms, and atypical mycobacteriosis [51]. These misdiagnoses may result in radical surgical interventions and the inappropriate administration of treatments, such as corticosteroids, that further complicate the histological diagnosis [32]. One of the primary contributors to delayed and inaccurate diagnosis in non-endemic regions is the limited familiarity of healthcare providers with leishmaniasis. A systematic review conducted by Sarfraz et al. showed that the average time from symptom onset to diagnosis was 4.5 years for leishmaniasis [52].

Besides the challenges posed by unfamiliarity and delayed presentation, the diagnosis of leishmaniasis is further complicated by the need for specific diagnostic techniques that differentiate species. Although the most widely used method for CL diagnosis is the identification of amastigotes in Giemsa-stained smears of excised tissue via light microscopy, this method of morphological examination cannot distinguish between Leishmania species. When performing microscopy, touch preparation smears are preferred over formalin-fixed paraffin-embedded tissue sections, as they provide better visualization of the kinetoplast [53]. This is a crucial feature that differentiates *Leishmania* from organisms that appear similar in tissue, like *Histoplasma capsulatum*. Moreover, although highly specific, it is not sufficiently sensitive [54]. This is imperative because deducing the specific Leishmania species plays a role in prognosis and management, since different species vary in their response to the currently available treatments [55]. To identify specific species, PCR, which has high specificity and sensitivity, can be used, but there has historically been a gap in standardizing the method, which is complex and expensive [56]. Alternatively, isoenzyme analysis of the cultured parasite can also be utilized to obtain species-level diagnosis, but it is time-consuming and necessitates isolates from culture [57]. Nonetheless, the use of advanced diagnostic methods is often limited in rural, non-endemic, and resource-limited areas, due to cost and limited access to trained personnel. In these settings, given the visual nature of CL, artificial intelligence (AI)-assisted image analysis and telemedicine offer scalable, cost-effective solutions. However, AI models require training on diverse, well-annotated datasets—a resource that has been limited in the development of AI algorithms for CL—while telemedicine depends on stable digital infrastructure [58].

## 7. Treatment of Cutaneous Leishmaniasis

The treatment approach of CL depends on factors such as species of *Leishmania*, disease severity, and geographic location. Conventional amphotericin B deoxycholate has been traditionally used to treat CL. However, lipid formulations of amphotericin B have been better tolerated, as the lipid formulation enhances drug delivery while reducing systemic toxicity [59]. The mechanism of action of liposomal amphotericin B is that it binds to ergosterol-like molecules in *Leishmania* cell membranes and results in parasite death through membrane disruption [60]. Limitations of this medication include high cost, the need for intravenous administration, which limits its accessibility in resource-poor settings, and its nephrotoxic potential, which requires renal function monitoring [60]. Emerging alternatives under investigation include oral amphotericin B formulations, which may alleviate the cost and need for intravenous administration [61].

Pentavalent antimonials such as sodium stibogluconate and meglumine antimoniate have been the first-line therapy in many endemic regions, and require either intramuscular or intravenous administration for 20–28 days [62]. However, they have limitations due to the potential for irreversible toxicity and variable effectiveness, as there have been increasing reports of resistance, particularly in India and the Middle East [63]. The mechanism of action of antimonials is the inhibition of *Leishmania* enzymes and inducing oxidative stress in the parasite [64].

Azole antibiotics have also been used in the treatment of CL, and their mechanism of action is to block the ergosterol synthesis of Leishmania parasites [65]. One of the key advantages of azoles is their ability to be administered orally, making them more accessible in resource-poor settings like refugee camps, while also offering fewer side effects [66].

Topical therapies are typically reserved for localized CL cases where the lesions are small, uncomplicated, and located away from the face or joints [67]. Some topical therapies include paromomycin, cryotherapy with liquid nitrogen, and thermotherapy [68].

Moreover, in 2014, oral miltefosine was approved by the FDA for the treatment of CL in adults and adolescents [69]. It is particularly effective against *L. braziliensis* and *L. panamensis*, though efficacy varies by region [70,71]. Its common side effects include gastrointestinal symptoms, and it is contraindicated in pregnant women [72]. Miltefosine works by affecting the parasite’s phospholipid metabolism and inducing apoptosis-like cell death [73].

Furthermore, immunotherapy for CL includes cytokine-based therapies such as interferon-γ therapy, which enhances macrophage activation and promotes parasite killing, and IL-12 therapy, which stimulates Th1 immune responses and promotes resistance to infection [74]. There are also therapeutic vaccines such as using killed parasites or DNA-based strategies to induce immunity. However, they are still under development [75]. Additionally, there are checkpoint inhibitors and immune modulators that enhance T-cell responses [76]. Lastly, experimental methods that are being explored include dendritic cell-based therapies and TLR agonists to stimulate innate immunity [77].

## 8. Integrated Approaches and Future Directions

Since the World Health Organization (WHO) considers Leishmania to be endemic in the United States, it should not be exclusively regarded as a tropical neglected disease. This inaccurate classification may lead clinicians to overlook the diagnosis, thereby delaying diagnostic evaluation and treatment. Consequently, when the diagnosis is confirmed, reporting should be required at the local and national levels, as well as to international health agencies. As of now, Texas is the only state in the United States that identifies leishmaniasis as a reportable disease, and has been doing so since 2007 [78]. If more states and countries mandated leishmaniasis reporting to health agencies, the geographic distribution, host species characteristics, climate change contributions, risk factors, and transmission would be better understood, and able to enhance surveillance and guide public health initiatives [79].

Targeted training programs for medical students and dermatologists in non-endemic countries are essential to ensure timely diagnoses. Since 2014, the WHO and the Open University of Catalonia have been providing interactive online training on the management of CL [80]. Additionally, the WHO previously appointed the Dermatology Department of La Rabta Hospital in Tunisia as a collaborating center to create training programs and develop guidelines for the management of CL [81]. In 2022, the WHO launched a strategic framework for the integrated control and management of skin-related neglected tropical diseases (skin NTDs), including leishmaniasis, detailing goals for 2021–2030 [82]. Countries were encouraged to incorporate milestones and targets to improve the control and management of skin NTDs [82]. Holistic implementation strategies proposed include skin screenings, training healthcare workers and volunteers in identification and diagnosis, implementing teledermatology and mHealth tools, and using common health information systems to integrate reporting, helping to streamline real-time data collection and analysis [82]. To further bolster surveillance systems, web-based, country-level, and mobile apps may enable data entry by states, health facilities, and individuals [83]. Additionally, fostering global collaboration through the establishment of research networks similar to ECLIPSE, a global health research program that strives to improve physical and mental health outcomes for people with CL, may help to combat emerging CL dermatological challenges through culturally sensitive community engagement [84].

## 9. Conclusions

CL is a significant and growing global health challenge, driven by increasing globalization, international travel, large-scale population movements such as migration and refugee displacement, ongoing conflicts, humanitarian crises, and the exacerbating effects of climate change on vector distribution. The disease, which disproportionately affects impoverished communities, is further compounded by insufficient healthcare expertise in non-endemic areas, the limited availability of diagnostic tools, and a general lack of awareness. Addressing this complex issue requires a multifaceted approach that includes targeted training programs for healthcare providers and volunteers in non-endemic regions, comprehensive screening protocols for at-risk populations, and the adoption of technologies for diagnosis, treatment, and surveillance. Integrated international reporting and comprehensive surveillance systems are essential to monitor disease trends and ensure appropriate policy changes and adequate resource allocation.

## Data Availability

No new data were created or analyzed in this study.

## Abbreviations

The following abbreviations are used in this manuscript:

CL	Cutaneous leishmaniasis
FDA	Food and Drug Administration
IFN-γ	Interferon-γ
IL-12	Interleukin-12
LCL	Localized cutaneous leishmaniasis
LMICs	Low- and middle-income countries
NTD	Neglected tropical diseases
PCR	Polymerase chain reaction
Th1	T-helper 1
Th2	T-helper 2
TLR	Toll-like receptor
WHO	World Health Organization
